# A Nomogram to Predict Long-Term Survival Outcomes of Patients Who Undergo Pneumonectomy for Non-small Cell Lung Cancer With Stage I-IIIB

**DOI:** 10.3389/fsurg.2021.604880

**Published:** 2021-04-29

**Authors:** Lei-Lei Wu, Wu-Tao Chen, Xuan Liu, Wen-Mei Jiang, Yang-Yu Huang, Peng Lin, Hao Long, Lan-Jun Zhang, Guo-Wei Ma

**Affiliations:** ^1^Department of Thoracic Surgery, Shanghai Pulmonary Hospital, Tongji University School of Medicine, Shanghai, China; ^2^Sun Yat-sen University Cancer Center, State Key Laboratory of Oncology in South China, Collaborative Innovation Center for Cancer Medicine, Guangzhou, China; ^3^Zhongshan School of Medicine, Sun Yat-sen University, Guangzhou, China

**Keywords:** pneumonectomy, non-small cell lung cancer, nomogram, cancer-specific survival, stage I-IIIB

## Abstract

**Background:** In this study, we aim to establish a nomogram to predict the prognosis of non-small cell lung cancer (NSCLC) patients with stage I-IIIB disease after pneumonectomy.

**Methods:** Patients selected from the Surveillance, Epidemiology, and End Results (SEER, *N* = 2,373) database were divided into two cohorts, namely a training cohort (SEER-T, *N* = 1,196) and an internal validation cohort (SEER-V, *N* = 1,177). Two cohorts were dichotomized into low- and high-risk subgroups by the optimal risk prognostic score (PS). The model was validated by indices of concordance (C-index) and calibration plots. Kaplan-Meier analysis and the log-rank tests were used to compare survival curves between the groups. The primary observational endpoint was cancer-specific survival (CSS).

**Results:** The nomogram comprised six factors as independent prognostic indictors; it significantly distinguished between low- and high-risk groups (all *P* < 0.05). The unadjusted 5-year CSS rates of high-risk and low-risk groups were 33 and 60% (SEER-T), 34 and 55% (SEER-V), respectively; the C-index of this nomogram in predicting CSS was higher than that in the 8th TNM staging system (SEER-T, 0.629 vs. 0.584, *P* < 0.001; SEER-V, 0.609 vs. 0.576, *P* < 0.001). In addition, the PS might be a significant negative indictor on CSS of patients with white patients [unadjusted hazard ration (HR) 1.008, *P* < 0.001], black patients (unadjusted HR 1.007, *P* < 0.001), and Asian or Pacific Islander (unadjusted HR 1.008, *P* = 0.008). In cases with squamous cell carcinoma (unadjusted HR 1.008, *P* < 0.001) or adenocarcinoma (unadjusted HR 1.008, *P* < 0.001), PS also might be a significant risk factor.

**Conclusions:** For post-pneumonectomy NSCLC patients, the nomogram may predict their survival with acceptable accuracy and further distinguish high-risk patients from low-risk patients.

## Introduction

Lung cancer remains the most common type of cancer, as it contributed to one quarter of all cancer-related deaths in 2019. Overall, 13% of estimated new cancer cases in men and women are lung cancer, which is responsible for ~24% of estimated new deaths among the 10 leading cancer types; lung cancer also has one of the lowest 5-year survival rates at 19% (1). Multiple therapy options are available for patients with non-small cell lung cancer (NSCLC), such as surgery, chemotherapy, radiation, and targeted therapy (2–7). Previous studies revealed that some factors might have an effect on prognoses, such as the lymph nodes ratio (LNR), pleura invasion, and surgical approaches (8–10).

Pneumonectomy, which was first performed by Macewen in 1895, has now become a common procedure in thoracic surgery and has been applied to massive and centrally located carcinoma that cannot be completely resected by lobectomy or lesser resection (11). A research reported that the 30-day mortality rate of patients with pneumonectomy was 16–20% (12). With respect to the side of pneumonectomy, a retrospective study has suggested that right pneumonectomy patients with stage I and II NSCLC had a higher risk in the overall survival, induced partly by the physiological importance of the right lung (13). However, the 5-year overall survival rate of patients receiving pneumonectomy was 32%, which was not satisfying (14).

Nomogram, which is a graphical calculating device, has been widely applied in clinical studies to predict prognosis based on relevant clinical characteristics (15, 16). Consisting of scales for each variable, nomogram provides clinical doctors with a convenient and effective way to estimate risk and make decisions. For patients after pneumonectomy, researchers paid more attention to the perioperative complications and perioperative death rate. Recently, some researchers constructed nomograms to predict long-term survival outcomes of NSCLC patients with pneumonectomy based on hematological indicators (17, 18).

Of note, the impact of adjuvant therapy on patients undergoing pneumonectomy remains unclear and controversial. An international adjuvant lung cancer trial in 2004 demonstrated an improved survival among patients with completely resected NSCLC (*P* < 0.03) (19); however, pneumonectomy patients did not have a survival benefit from adjuvant chemotherapy. In addition, the benefit of post-operative radiotherapy (PORT) in pN2 NSCLC patients has been established in a large-scale retrospective study (3). Therefore, this study aims to develop a nomogram for predicting prognosis of NSCLC patients who undergo pneumonectomy. We hope that this prognostic model may provide some information for clinicians to decide on the strategy of follow-up management and adjuvant therapy in patients with stage IA-IIIB NSCLC.

## Materials and Methods

### Patients

**Figure 1:**
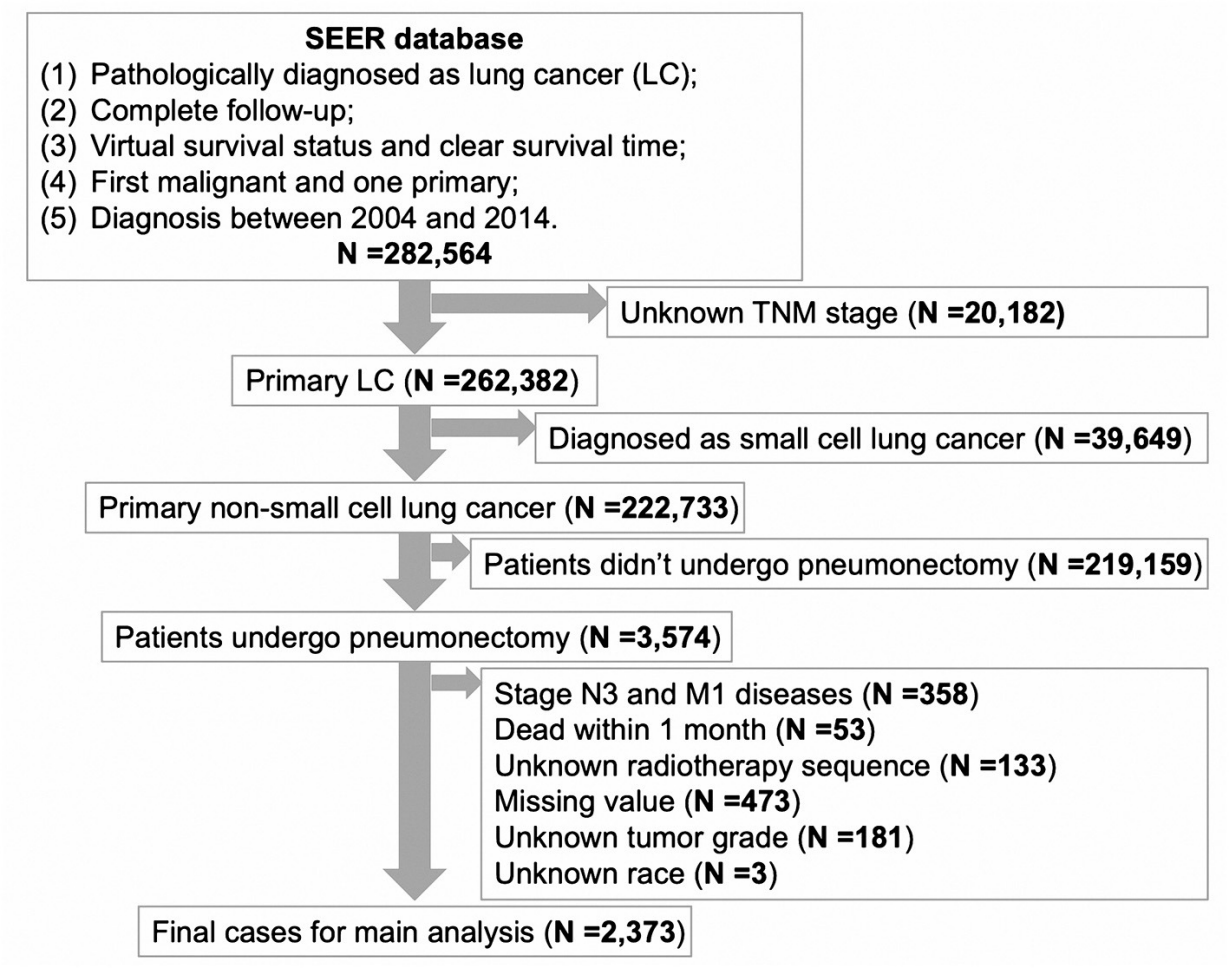
Flow diagram of this cohort study.

We identified eligible patients from the Surveillance, Epidemiology, and End Results (SEER) database. Cases were identified using the NSCLC participant data file from the SEER, which contain deidentified patients level data that do not identify hospitals, health care providers, or patients. Institutional review board approval was waived by the Ethics Committee of Sun Yat-sen University Cancer Center. Tumor, nodes, and metastasis (TNM) stages were determined by the classification reported in the 8th American Joint Committee on Cancer (AJCC) Staging Manual. We enrolled a total of 2,373 patients from the SEER database. Patients were randomly divided into two cohorts: the training cohort (SEER-T, *N* = 1,196) and an internal validation cohort (SEER-V, *N* = 1,177), as shown in Figure 1. All patients recruited for this study met the following criteria: (1) patients underwent pneumonectomy; (2) patients had pathological confirmation of NSCLC; and (3) patients with stage IA-IIIB disease, and IIIB only included T3-4N2M0 tumors. Patients were excluded if they met these criteria: (1) patients with another malignant tumor; (2) patients with an incomplete follow-up; (3) patients who died within 1 month after surgery; and (4) patients under 18 years old.

### Follow-Up

The median follow-up time for patients in the SEER database was 70.0 months. Follow-ups were performed by office visits or telephone interviews. Cancer-specific survival (CSS) was defined as the interval between the time of resection to the time of death from cancer.

### Statistical Methods

A comparison of categorical variables was conducted by Pearson's Chi-square test. A multivariate analysis was performed to evaluate the influences of gender, age, maximum tumor diameter, laterality of pneumonectomy, grade, TNM stage, primary tumor location, race, pleural invasion, LNR, chemotherapy, radiotherapy, and pathological subtypes on CSS. A two-sided *P* < 0.05 was considered statistically significant. The most valuable prognostic factors identified by the univariate analysis were confirmed by a multivariate Cox regression analysis and excluded other confounding factors that might have affected the survival. In addition, Kaplan–Meier analysis and the log-rank tests were used to compare survival curves between groups. Cases were censored at death or at the end of follow-up. The selection of CSS as a primary clinical end point was considered the most clinically relevant. We adopted a model development and validation approach, and used a randomized method to extract trained and validated data sets to maintain a transparent report of the multivariate predictive model on individual prognostic guidelines.

**Figure 2:**
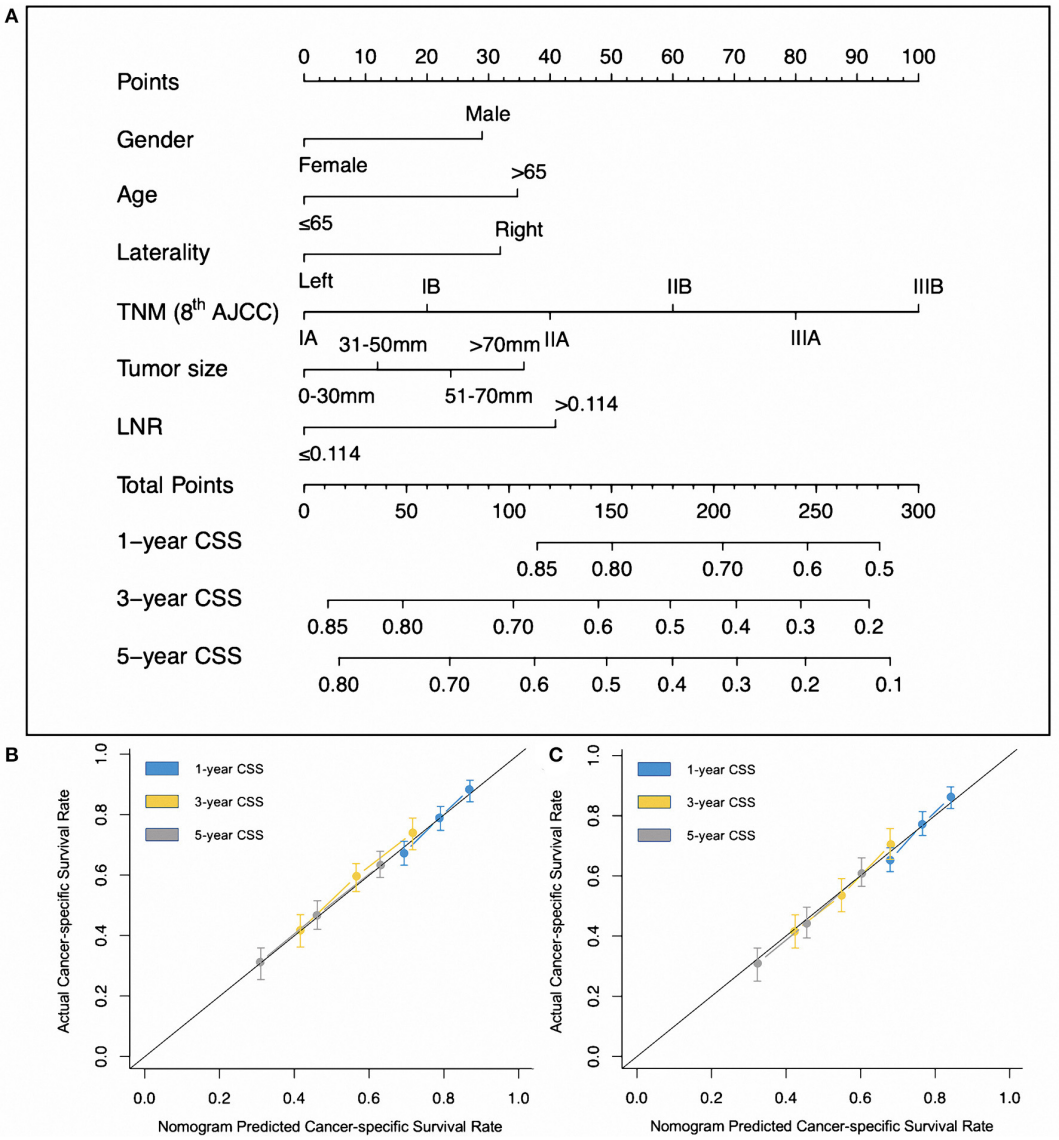
**(A)** Post-operative prognostic nomogram for patients after pneumonectomy with stage I-IIIB non-small-cell lung cancer. **(B)** Calibration curves for predicting cancer-specific survival of patients at each time point in the training cohort. **(C)** Calibration curves for predicting cancer-specific survival of patients at each time point in the internal validation cohort.

Patients' demographics and clinical characteristics were reported for the training cohort. We constructed a nomogram based on six factors: gender, age, laterality of pneumonectomy, LNR, tumor size, and TNM stage. The model for CSS was constructed using the linear predictor of the finalized model, which was derived from the training data set. We regarded the prognostic score (PS) derived from the nomogram as an evaluation tool. The PS depended on the contribution of each variable to CSS from the cox regression model we constructed. The variables which contributed to CSS was matched to the horizontal axis at the top of Figure 2A, and the PS each variable presented was added up as total points. Two cohorts (SEER-T, SEER-V) were split into low- and high-risk subgroups by the optimal PS among non-living patients in the training cohort, and a risk score cutoff was defined to classify patients in the validation cohorts. The optimal cutoff point of the PS and LNR was calculated using the “survminer” package in R 3.6.1 software (https://www.r-project.org/). Data were analyzed using R 3.6.1 and SPSS software (version 24; IBM Corp., USA). Indices of concordance (C-index) were generated for the discrimination of the multivariable prognostic model.

## Results

### Patient Characteristics

Overall, 2,373 patients were identified from the SEER database, including 1,196 patients in the training cohort (SEER-T) and 1,177 patients in the internal validation cohort (SEER-V).

In the SEER-T and SEER-V cohorts, a large proportion of patients was under the age of 65 years (60.9% in SEER-T and 64.5% in SEER-V). Male patients were predominant in both cohorts (67.6% in the SEER-T and 66.0% in the SEER-V). A tumor located in the left lung was quite common and had a distribution of 57.9% in the SEER-T and 59.0% in the SEER-V. The median survival time was 56.0 months for patients in the SEER-T and 48.0 months in the SEER-V. The clinical information of patients from the SEER database is shown in Table 1.

**Table 1:** Patients characteristics from SEER database.

**Cohort**	**SEER-T**	**SEER-V**	***P*-value**
Total	1,196	1,177	
Sex (%)			0.451
Male	808 (67.6)	777 (66.0)	
Female	388 (32.4)	400 (34.0)	
Age (%)			0.075
≤ 65	728 (60.9)	759 (64.5)	
>65	468 (39.1)	418 (35.5)	
Race (%)			0.851
American Indian/Alaska Native	4 (0.3)	6 (0.5)	
Asian or Pacific Islander	59 (4.9)	64 (5.4)	
Black	102 (8.5)	97 (8.2)	
White	1,031 (86.2)	1,010 (85.8)	
Laterality (%)			0.614
Left	693 (57.9)	695 (59.0)	
Right	503 (42.1)	482 (41.0)	
Grade (%)			0.130
I	49 (4.1)	64 (5.4)	
II	441 (36.9)	458 (38.9)	
III	653 (54.6)	617 (52.4)	
IV	53 (4.4)	38 (3.2)	
TNM (%)			0.115
IA	56 (4.7)	60 (5.1)	
IB	101 (8.4)	85 (7.2)	
IIA	49 (4.1)	63 (5.4)	
IIB	305 (25.5)	261 (22.2)	
IIIA	528 (44.1)	523 (44.4)	
IIIB	157 (13.1)	185 (15.7)	
Tumor size (%)			0.935
0–30 mm	243 (20.3)	229 (19.5)	
31–50 mm	405 (33.9)	406 (34.5)	
51–70 mm	286 (23.9)	277 (23.5)	
>70 mm	262 (21.9)	265 (22.5)	
Tumor location (%)			0.950
Main bronchus	596 (49.8)	586 (49.8)	
Upper lobe	46 (3.8)	43 (3.7)	
Middle lobe	300 (25.1)	310 (26.3)	
Lower lobe	110 (9.2)	110 (9.3)	
Overlapping	112 (9.4)	100 (8.5)	
NOS	32 (2.7)	28 (2.4)	
Pleural invasion (%)			0.732
No	1,103 (92.2)	1,080 (91.8)	
Yes	93 (7.8)	97 (8.2)	
Histology types (%)			0.805
SCC	642 (53.7)	628 (53.4)	
Adeno	368 (30.8)	379 (32.2)	
LCC	47 (3.9)	37 (3.1)	
SC	7 (0.6)	6 (0.5)	
Other	75 (6.3)	79 (6.7)	
Unknown	57 (4.8)	48 (4.1)	
LNR (%)			0.115
≤ 0.114	669 (55.9)	697 (59.2)	
>0.114	527 (44.1)	480 (40.8)	
Chemotherapy (%)			0.421
No	601 (50.3)	571 (48.5)	
Yes	595 (49.7)	606 (51.5)	
Radiation (%)			0.341
No	953 (79.7)	938 (79.7)	
Post-operative radiotherapy	164 (13.7)	146 (12.4)	
Pre-operative radiotherapy	79 (6.6)	93 (7.9)	

*SEER, Surveillance, Epidemiology, and End Results; LNR, lymph node ratio; TNM, tumor, nodes, and metastasis; SCC, squamous cell carcinoma; Adeno, adenocarcinoma; LCC, large cell carcinoma; SC, sarcomatoid carcinoma*.

### Univariate and Multivariate Analyses

The results of univariate and multivariate analyses are presented in Table 2. In the univariate analysis, female sex (vs. male; *P* = 0.003), left-side pneumonectomy (vs. right-side; *P* = 0.008), and younger age (≤65 vs. >65 years; *P* < 0.001) appeared as protective factors. Among other factors, grade (*P* < 0.001), TNM stage (*P* < 0.001), tumor size (*P* = 0.006), and LNR (*P* < 0.001) had an impact on survival, while information related to race, tumor location, histological types, chemotherapy record, radiotherapy record, and pleural invasion were considered insignificant. In the multivariate analysis, after adjustment for other factors, sex, age, laterality, tumor size, LNR, and TNM stage were established as independent factors and thus used to construct the nomogram.

**Table 2:** Univariate and multivariate analyses in SEER-T cohort.

**Variable**	**Univariate analysis**		**Multivariate analysis**	
	**HR (95%CI)**	***P*-value**	**HR (95%CI)**	***P*-value**
Sex		0.002		0.006
Male	Reference		Reference	
Female	0.764 (0.640–0.911)	0.003	0.779 (0.652–0.93)	0.006
Age		<0.001		<0.001
≤ 65	Reference		Reference	
>65	1.33 (1.130–1.560)	<0.001	1.353 (1.147–1.595)	<0.001
Laterality		0.008		0.001
Left	Reference		Reference	
Right	1.240 (1.060–1.460)	0.008	1.318 (1.119–1.551)	0.001
Grade		<0.001		
I	Reference			
II	1.940 (1.130–3.340)	0.016		
III	2.320 (1.360–3.960)	0.002		
IV	2.850 (1.520–5.350)	0.001		
TNM stage		<0.001		0.003
IA	Reference		Reference	
IB	1.180 (0.618–2.270)	0.610	0.911 (0.464–1.789)	0.786
IIA	1.910 (0.965–3.760)	0.0631	1.394 (0.671–2.897)	0.374
IIB	1.830 (1.030–3.230)	0.0382	1.283 (0.695–2.369)	0.425
IIIA	2.860 (1.640–4.980)	<0.001	1.824 (0.983–3.384)	0.057
IIIB	3.300 (1.850–5.890)	<0.001	2.016 (1.043–3.896)	0.037
Tumor size		0.006		0.039
0–30 mm	Reference		Reference	
31–50 mm	1.530 (1.190–1.960)	<0.001	1.349 (1.031–1.766)	0.029
51–70 mm	1.540 (1.180–2.010)	0.001	1.153 (0.858–1.551)	0.345
>70 mm	2.100 (1.610–2.730)	<0.001	1.454 (1.067–1.98)	0.018
LNR		<0.001		<0.001
≤ 0.114	Reference		Reference	
>0.114	1.600 (1.360–1.880)	<0.001	1.421 (1.180–1.712)	<0.001
Tumor location		0.288		
Main bronchus	Reference			
Upper lobe	0.889 (0.558–1.410)	0.619		
Middle lobe	1.220 (1.010–1.490)	0.041		
Lower lobe	1.190 (0.899–1.580)	0.224		
Overlapping	1.210 (0.915–1.610)	0.179		
NOS	1.150 (0.686–1.940)	0.592		
Race		0.508		
American Indian/Alaska Native	Reference			
Asian or Pacific Islander	NA	0.989		
Black	NA	0.989		
White	NA	0.989		
Pleural invasion		0.997		
No	Reference			
Yes	1.000 (0.736–1.360)	0.997		
Histology types		0.212		
SCC	Reference			
Adeno	1.050 (0.878–1.260)	0.585		
LCC	1.520 (1.050–2.220)	0.028		
SC	0.502 (0.125–2.020)	0.332		
Other	1.150 (0.814–1.610)	0.437		
Unknown	0.855 (0.576–1.270)	0.434		
Chemotherapy		0.199		
No	Reference			
Yes	0.900 (0.766–1.060)	0.199		
Radiation		0.110		
No	Reference			
Post-operative	1.250 (1.000–1.550)	0.047		
Pre-operative	1.180 (0.865–1.610)	0.296		

*SEER, Surveillance, Epidemiology, and End Results; LNR, lymph node ratio; HR, hazard ratio; CIs, confidence intervals; TNM, tumor, nodes, and metastasis; SCC, squamous cell carcinoma; Adeno, adenocarcinoma; LCC, large cell carcinoma; SC, sarcomatoid carcinoma*.

### Nomogram Construction and Survival Analysis

**Figure 3:**
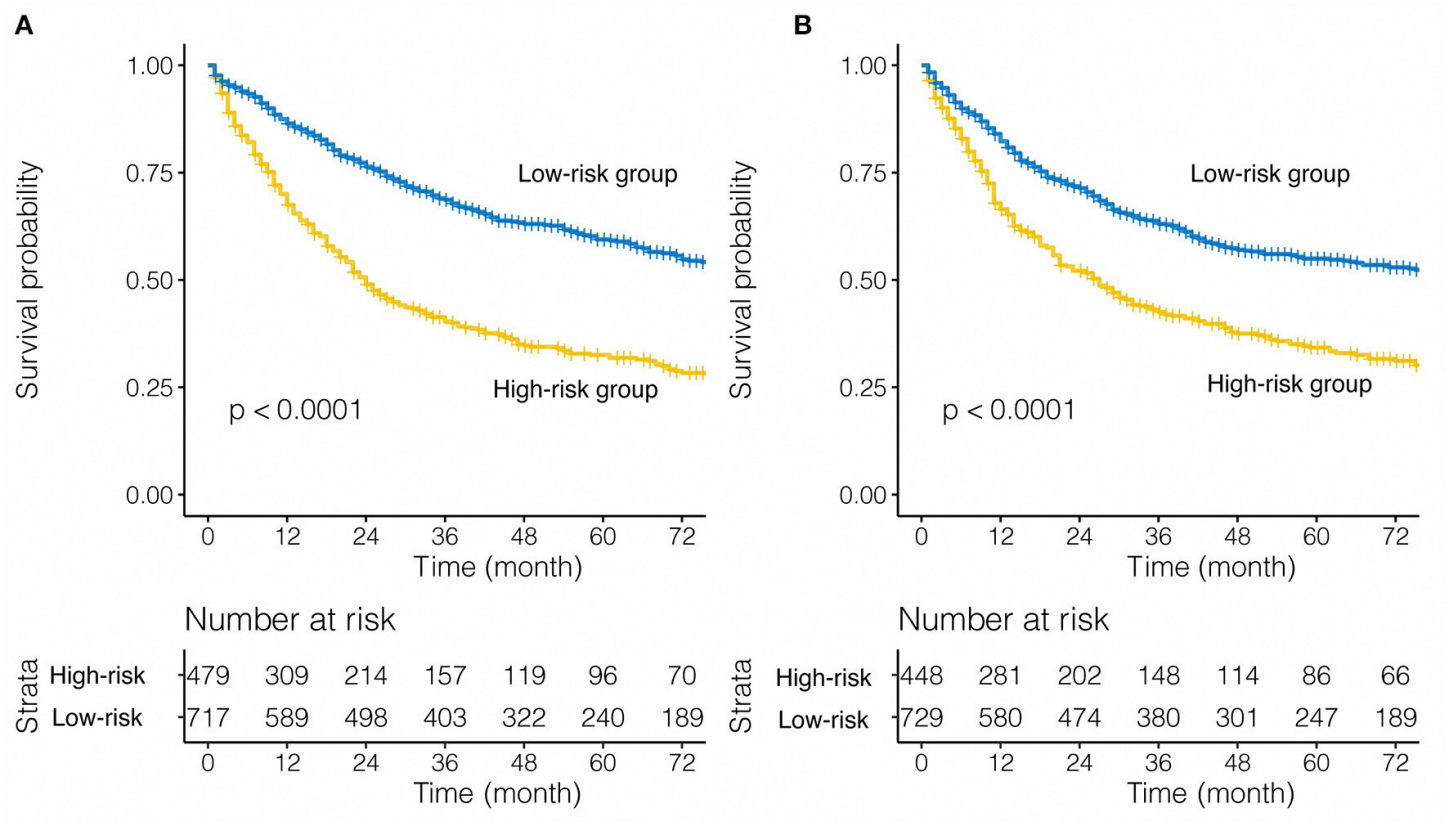
Stratified effect of the nomogram on the training cohort **(A)** and internal validation cohort **(B)**.

Considering the statistical significance of the risk factors in the multivariate analysis, we developed a nomogram to predict the prognosis of patients undergoing pneumonectomy using eligible variables (sex, age, laterality, tumor size, LNR, and TNM stage) (Figure 2A). The calibration plot showed good concordance with respect to prediction accuracy in both SEER-T and SEER-V with a C-index of 0.629 and 0.609, respectively (Figures 2B,C). The constructed prognostic score is shown in the Table 3. The “survminer” package in R was applied to determine the optimal cutoff value of the PS. Thus, the training data were divided into a high-risk group (*N* = 479) and a low-risk group (*N* = 717) by setting the cutoff point at 165. The C-index of 0.629 indicated an acceptable discrimination ability of this model. The Kaplan-Meier curve showed that patients in the high-risk group had significantly poor survival (Figure 3A, unadjusted HR = 2.219; 95% CI 1.887–2.609, *P* < 0.001). In view of the survival probability, the 1-year CSS was 68% in the high-risk group vs. 86% in the low-risk group, the 3-year CSS was 40 vs. 69%, and the 5-year CSS was 33 vs. 60%. The median survival time in the high- and low-risk group was 24.0 months and 108.0 months, respectively.

**Table 3:** Point assignment.

**Variable**	**Prognostic score**
Sex	
Male	29
Female	0
Age	
≤ 65	0
>65	35
TNM stage	
IA	0
IB	20
IIA	40
IIB	60
IIIA	80
IIIB	100
Laterality	
Left	0
Right	32
Tumor size	
0–30 mm	0
31–50 mm	12
51–70 mm	24
>70 mm	36
LNR	
≤ 0.114	0
>0.114	41
Risk stratification	
High risk	>165
Low risk	≤ 165

*LNR, lymph node ratio; TNM, tumor, nodes, and metastasis*.

### Validation of the Nomogram

For the internal validation cohort, the Kaplan-Meier curve demonstrated poorer survival in the high-risk group (Figure 3B, SEER-V: unadjusted HR = 1.780; 95% CI 1.515–2.092, *P* < 0.001) than low-risk group. The C-index of the validation cohort was acceptable and reached 0.609. For SEER-V, the 1-year CSS was 66 vs. 82%, the 3-year CSS was 43 vs. 63%, and the 5-year CSS was 34 vs. 55% (high-risk vs. low-risk). The median survival time was 27.0 months in the high-risk group and 87.0 months in the low-risk group.

We found that our prognostic model of nomogram had a better predictive ability than that in the 8th AJCC TNM staging system (SEER-T, C-index = 0.629 vs. 0.584, respectively, *P* < 0.001; SEER-V, C-index = 0.609 vs. 0.576, respectively, *P* < 0.001).

### Subgroup Analysis of Nomogram

**Figure 4:**
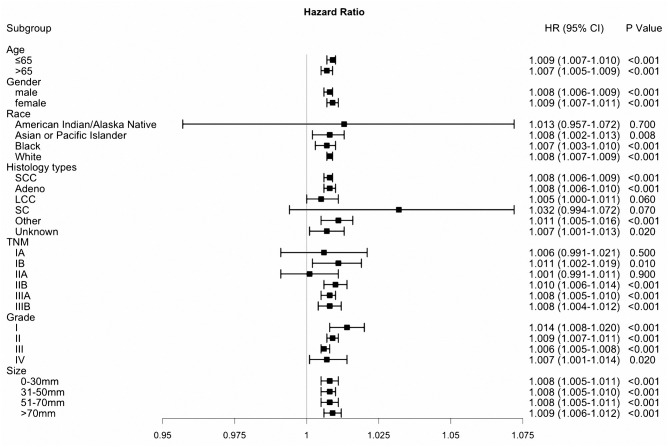
Subgroup analysis based on the prognostic score.

**Figure 5:**
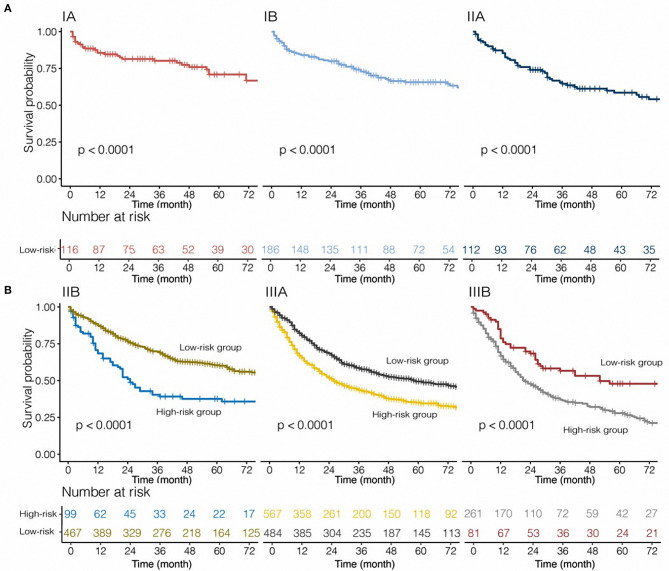
Risk group stratification within each stage for tumor, nodes, and metastasis [**(A)** patients with stage IA-IIA non-small-cell lung cancer; **(B)** patients with stage IIB-IIIB non-small-cell lung cancer] in this cohort.

Based on the 8th TNM stage, patients with IA-IIIB cancer stage were included in our study. Categorized by pathological TNM stage, the model was applied to all patients from the SEER database. In stage IB, IIB, IIIA, and IIIB, PS performed as a risk prognostic factor (all HR >1, all *P* < 0.05, Figure 4). However, based on the Kaplan–Meier analysis and log-rank tests, we found that patients with stage IA, IB, and IIA disease belonged to the low-risk group (Figure 5). The nomogram had obvious stratified effect on CSS of patients with stage IIB, IIIA, and IIIB (all *P* < 0.01, Figure 5). As for different race/ethnicity, we suggested that the PS might have a negative effect on CSS of patients with white patients, black patients, and Asian or Pacific Islander (Figure 4). In addition, PS had poor prognosis in patients with squamous cell carcinoma (SCC) or adenocarcinoma.

## Discussion

This study aimed to develop a nomogram to predict the CSS of patients with NSCLC who underwent pneumonectomy. Altogether, six variables were considered. After performing univariate and multivariate analyses on the SEER-T cohort, independent prognostic factors such as sex, age, laterality, tumor size, LNR, and TNM stage were identified, while factors such as grade, tumor location, pathology, chemotherapy, radiotherapy, pleural infiltration, and race were excluded due to statistical insignificance. The nomogram for CSS was constructed using the previously stated eligible variables and achieved an acceptable C-index of 0.629 (95% CI 0.627–0.630). A prognostic score was calculated for each patient using the model and was dichotomized by setting the cutoff point to 165. The high- and low-risk groups were derived from the SEER-T based on the dichotomized PS, which showed good distinction with ~20% increase in the 1, 3, 5-year survival rates in the low-risk group compared with the high-risk group. In order to verify this result, the model was applied to the SEER-V, which showed good distinction and achieved acceptable discrimination with a C-index of 0.609 (95% CI 0.607–0.610). The results demonstrated that this model could be used well in another internal validation cohort. In addition, the model fitted well in patients with stage IIB, IIIA, and IIIB disease and showed evident differences in the 1, 3, 5-year CSS rates in the low-risk group compared with the high-risk group. We suggested that this model, which was based on six indicators, could distinguish high-risk post-operative patients from low-risk patients. Nomogram was used to calculate individual risk scores for cancer prognoses. Therefore, it is possible to predict the prognosis of an eligible patient, thus providing more direction for individualized follow-up management and therapy. We hope to build an easy model by using commonly obtained patient information. Eventually, six meaningful indicators were selected using univariate and multivariate analyses of the training cohort. We constructed a nomogram based on the above six factors and successfully identified high-risk and low-risk populations in the training and validation cohorts. Our model had a significant impact on patient differentiation (Figure 3), because the C index for predicting CSS rates reached 0.629 (SEER-T), and 0.609 (SEER-V) in the training and validation cohorts, respectively. In terms of the clinical application, these indicators can be easily assessed. Clinicians could use the above information and our model to calculate scores of NSCLC patients with pneumonectomy, and give patients advice on whether adjuvant therapy and the strategy of follow-up management.

The impact of adjuvant chemotherapy on patients undergoing pneumonectomy remains unclear and controversial. An international adjuvant lung cancer trial in 2004 demonstrated an improved survival among patients with completely resected NSCLC (*P* < 0.03) (19); however, pneumonectomy patients did not have a survival benefit from adjuvant chemotherapy. Similarly, in our study, chemotherapy did not act as a significant prognostic factor on CSS. In addition, PORT was not identified as an independent prognostic factor in this study. The benefit of PORT in pN2 NSCLC patients has been established in a large-scale retrospective study (3). In our study, however, PORT was more of a risk factor in the univariate analysis of the pneumonectomy cohort (HR = 1.250; 95% CI 1.000–1.550, *P* = 0.047). The LNR was regarded as a valuable prognostic factor in NSCLC patients, although it is not well-understood in patients who undergo pneumonectomy. Taylor et al. claimed that a higher LNR was correlated with a worse prognosis and a higher rate of local recurrence and vice versa (20). However, there were only 88 patients with pneumonectomy included in their study. Besides, Han et al. explained that the LNR could predict the survival probability of pIIIA-N2 patients after surgery and post-operative chemotherapy (21). There were 2804 patients (including 27 patients with pneumonectomy) for main analyses. Chiappetta et al. also suggested that a high LNR was a risk prognostic factor, and was related to a poor survival outcome. Their findings were based on a multicenter analysis (22). Nevertheless, in the above-mentioned studies, pneumonectomy was not discussed separately. In our study, the prognostic impact of LNR was similar to their results that a high LNR correlated with poor prognosis for patients after pneumonectomy. In addition, the effect of LNR on pneumonectomy was shown in Figure 3 and Table 2.

The impact of right or left pneumonectomy on prognosis remains controversial. Riquet et al. discovered that the overall survival after pneumonectomy among the 10-year survivors (*N* = 250) could not be meaningfully distinguished by the laterality of tumor/surgery (14). Similarly, in 2019, Yang et al. reported that right-sided or left-sided pneumonectomy after induction chemotherapy was not beneficial for long-term survival (23). Wang et al. also analyzed the data of 100 cases with pneumonectomy and found that the right- or left-side resection didn't give a long-term survival benefit for those patients (24). However, according to the study conducted by Dhanasopon et al., right pneumonectomy patients had worse prognosis compared to left pneumonectomy patients (25). Their study included 79,953 patients, 4,245 of whom underwent pneumonectomy. Simón et al. reported that stage I and II NSCLC patients had better survival with left pneumonectomy based on data of 1,475 patients (including 421 patients after pneumonectomy) (13). The results of our study were similar to those of Dhanasopon et al. and Simón et al. and showed that right-sided pneumonectomy was an independent risk factor for CSS (Table 2).

Nevertheless, several drawbacks exist in this study. Above all, this study was restricted by its retrospective design. Above six independent prognostic factors were derived from a retrospective study. Thus, a prospective study is required to fully validate the model in the clinical application. Confounding errors and biases were limited to an extent by the univariate and multivariate analyses, yet they were inevitable due to the disproportionately distributed variables, such as the TNM stage, races, and tumor location. Hopefully, with a larger sample size, the problem of disproportionate distribution could be solved. Moreover, due to the nature of the SEER database, we could not acquire complete information on the chemotherapy, radiotherapy, and perioperative complications. Therefore, it remained unclear whether chemotherapy, radiotherapy, or perioperative complications had an impact on long-term survival in this pneumonectomy cohort study. Further studies are needed with elaborate medical records to assess the impact of above these factors.

## Conclusion

We developed a nomogram for patients with NSCLC undergoing pneumonectomy, which achieved acceptable discrimination in the internal validation cohort. For eligible patients who would be classified in the high-risk group, supplemental individualized follow-up management and therapy should be provided in order to achieve better survival. The study also reported that right lung pneumonectomy was a high-risk prognostic factor compared to the left lung pneumonectomy. In addition, multicenter prospective studies and large number of cases are needed to confirm our findings.

## Data Availability Statement

Publicly available datasets were analysed in this study. This data can be found here: https://seer.cancer.gov/.

## Ethics Statement

Ethical review and approval was not required for the study on human participants in accordance with the local legislation and institutional requirements. Written informed consent for participation was not required for this study in accordance with the national legislation and the institutional requirements.

## Author Contributions

G-WM and L-LW: conceptualization. L-LW, W-MJ, XL, Y-YH, and W-TC: data curation. W-TC, L-LW, and G-WM: formal analysis. G-WM, L-LW, and W-TC: methodology. G-WM, L-JZ, HL, and PL: project administration and supervision. L-LW, G-WM, and XL: validation. W-TC and L-LW: roles/writing—original draft. G-WM, L-LW, XL, and W-MJ: writing—review and editing. All authors contributed to the article and approved the submitted version.

## Conflict of Interest

The authors declare that the research was conducted in the absence of any commercial or financial relationships that could be construed as a potential conflict of interest.

## Abbreviations

NSCLC: non-small cell cancer
SEER: Surveillance, Epidemiology, and End Results
PS: prognostic score
LNR: lymph node ratio
HR: hazard ratio
CIs: confidence intervals
C-index: indices of concordance
SD: standard deviation
CSS: cancer-specific survival
AJCC: American Joint Committee on Cancer
TNM: tumor, nodes, and metastasis
PORT: post-operative radiotherapy
SCC: squamous cell carcinoma
Adeno: adenocarcinoma
LCC: large cell carcinoma
SC: sarcomatoid carcinoma.
